# FSP1 promotes the biofunctions of adventitial fibroblast through the crosstalk among RAGE, JAK2/STAT3 and Wnt3a/β‐catenin signalling pathways

**DOI:** 10.1111/jcmm.14518

**Published:** 2019-08-27

**Authors:** Caihua Fu, Ping Liu, Peilun Li, Wenhui Liu, Xianwei Huang, Yansheng Liang

**Affiliations:** ^1^ Department of Cardiology Jinan Central Hospital Affiliated Shandong University Jinan China; ^2^ Department of Cardiology The Second Hospital of Shandong University Jinan China; ^3^ Department of Cardiology Linyi People's Hospital Linyi China; ^4^ Department of Emergency First Affiliated Hospital of Xiamen University Xiamen China

**Keywords:** adventitial fibroblast, autophagy, FSP1, signalling pathway, siRNA‐FSP1

## Abstract

Emerging evidence indicates that fibroblast‐specific protein 1 (FSP1) provides vital effects in cell biofunctions. However, whether FSP1 influences the adventitial fibroblast (AF) and vascular remodelling remains unclear. Therefore, we investigated the potential role and action mechanism of FSP1‐mediated AF bioactivity. AFs were cultured and stimulated with FSP1 and siRNA‐FSP1 in vitro. Viability assays demonstrated that siRNA‐FSP1 counteracted AFs proliferative, migratory and adherent abilities enhanced with FSP1. Flow cytometry revealed that FSP1 increased AFs number in S phase and decreased cellular apoptosis. Contrarily, siRNA‐FSP1 displayed the contrary results. RT‐PCR, Western blotting and immunocytochemistry showed that FSP1 synchronously up‐regulated the expression of molecules in RAGE, JAK2/STAT3 and Wnt3a/β‐catenin pathways and induced a proinflammatory cytokine profile characterized by high levels of MCP‐1, ICAM‐1 and VCAM‐1. Conversely, FSP1 knockdown reduced the expression of these molecules and cytokines. The increased number of autophagosomes in FSP1‐stimulated group and fewer autophagic corpuscles in siRNA‐FSP1 group was observed by transmission electron microscope (TEM). Autophagy‐related proteins (LC3B, beclin‐1 and Apg7) were higher in FSP1 group than those in other groups. Conversely, the expression of p62 protein was shown an opposite trend of variation. Therefore, these pathways can promote AFs bioactivity, facilitate autophagy and induce the expression of the proinflammatory cytokines. Contrarily, siRNA‐FSP1 intercepts the crosstalk of these pathways, suppresses AF functions, restrains autophagy and attenuates the expression of the inflammatory factors. Our findings indicate that crosstalk among RAGE, STAT3/JAK2 and Wnt3a/β‐catenin signalling pathways may account for the mechanism of AF functions with the stimulation of FSP1.

## 1 INTRODUCTION

The conventional mindset is that the extravascular membrane only supports and nourishes blood vessels.^1^, ^2^, ^3^ Now confirmed, whether vascular inflammation, early intimal thickening or late fibrosis of the vessel wall after vascular injury, vascular adventitial fibroblasts (AF) have played a central role in vascular remodelling.^1^, ^2^, ^4^ Abundant evidence indicate that resident AF is directly involved in the vascular remodelling of atherosclerosis, hypertension, pulmonary hypertension and restenosis.^2^, ^4^

During the process of vascular proliferative pathology, AF secretes a variety of biological factors including fibroblast‐specific protein 1 (FSP1) to influence vascular remodelling.^1^, ^2^, ^4^, ^5^ As a member of the S100 calcium‐binding protein family and one of the markers of fibroblasts, FSP1 (also known as S100A4) was first cloned in fibroblasts and tumour cells.^6^ Recently, some researches demonstrate that FSP1 is not only closely related to the development of inflammation and tumour but also closely related to cardiovascular fibrosis and vascular remodelling.^7^, ^8^ In humans and animals, FSP1 markedly accumulates in restenotic region after coronary stent placement and intracranial aneurysms.^8^ FSP1 is secreted in a diversity of cells, binds to several targets and exerts extracellular and intracellular effects functions in regulating cell adhesion, proliferation, differentiation, motility, invasion, angiogenesis and inflammation maintaining 6, 9 by recruiting multiple membrane receptors (such as receptor for advanced glycation endproducts, RAGE) and activating NF‐kB, and other signalling pathways.^8^, ^9^ Instead, the deletion of FSP1 results in defective cell migration, absence of cell protection, cardiac dysfunction, apoptosis and necrosis, capillary density decrease and cell reduced in number.^10^, ^11^ However, no data are available for the specific role and functionary mechanism of FSP1 in AF biofunctions realization.

The most characteristic of FSP1 is that it works through RAGE outside the cell in a cytokine‐like manner.^12^, ^13^ FSP1 primarily activates RAGE and in turn enabled RAGE accounts for STAT3 activation to trigger vascular remodelling,^14^ otherwise, which was reversed by RAGE inhibition.^14^ Moreover, FSP1 is also the target gene for Wnt/β‐catenin and may interact with the signalling molecule via regulating β‐catenin phosphorylation and deactivation in cardiac fibrosis.^15^ FSP1 promoted cell proliferation by stimulating the Wnt/β‐catenin pathway in a RAGE‐dependent manner.^6^ Coincidentally, Wnt/β‐catenin can also transcriptionally regulate the activation of STAT pathway to modulate macrophage inflammatory responses and atherosclerosis.^16^ Thus, we have every reason to speculate FSP1 may induce the crosstalk among the RAGE, JAK/STAT and Wnt/β‐catenin pathways to achieve the corresponding cellular function in AFs. Though in vivo and in vitro the examples of interaction between RAGE and JAK/STAT or Wnt/β‐catenin pathways have been well documented in some cells, the crosstalk among Rage, JAK/STAT and Wnt/β‐catenin pathways in AFs has not been integratively elaborated. Moreover, the exact role of FSP1 in AF has not yet been described. In this study, we tried to investigate the molecular signalling mechanism of FSP1 affecting the biofunctions of AF in vitro.

As the RAGE’s ligands, FSP1 is involved in regulating autophagy.^12^ Autophagy is primarily charge of the degradation of ageing proteins and cytoplasmic organelles in cells.^17^ Recent investigations shed more light onto the detailed mechanisms of moderate autophagy can protect cells from cellular injury under stressful environment.^17^, ^18^ Recently, few individuals have studied the relationship between FSP1 and autophagy.^6^, ^12^ However, information about the function of autophagy in AFs in normal and pathological conditions is fragmentary. Therefore, this is the first time we have proposed whether FSP1 participates in autophagic regulation in AF cells.

Taken together, since the previous studies demonstrated that FSP1 activated RAGE and Wnt/β‐catenin, and Wnt/β‐catenin also interacted with STAT3, so we proposed a possible underlying molecular mechanism, whereby FSP1 induces the crosstalk among RAGE, JAK/STAT and Wnt/β‐catenin signalling pathways to modulate AFs bioactivity. In this work, to prove our hypothesis, we applied several signal molecules and autophagy modulators and FSP1‐siRNA to determine the involvement of these signals crosstalk to adjust the AFs functions of proliferation, migration, adhesion, apoptosis and autophagy.

## 2 MATERIALS AND METHODS

### 2.1 Materials

Mice were purchased from Shandong University Experimental Animal Center (Jinan, China). Primary antibodies for Western blotting analysis were purchased from CST (USA) as follows: S100A4 (D9F9D), JAK2 (D2E12), p‐JAK2 (Tyr1007) (D15E2), LC3B (D11), STAT3 (D1B2J), p‐STAT3 (Tyr705) (D3A7), Wnt3a (C64F2), β‐catenin (D10A8) and p‐β‐catenin (Tyr869) (D4A6). Antibodies from Abcam are as follows: TCF4 (ab185736), RAGE (ab3611), P4HA1 (ab244302), Collagen I (ab6308) and Collagen III (ab7778). PVDF membranes were purchased from Millipore Corporation. ECL detection reagents were from Amersham Biosciences. MTT and EdU were purchased from Beyotime. DAPI was purchased from Sigma‐Aldrich Corporation (St. Louis.). Fluorescent mounting medium was obtained from Dako Corporation. PrimeScript^™^ RT reagent kit with gDNA Eraser was purchased from Takara Bio Inc (TaKaRa). Electron microscope fixation liquid was purchased from (solarbio). Other reagents were obtained from Invitrogen and Zhongshan Golden Bridge Biotechnology.

### 2.2 siRNA‐FSP1 Preparation

The siRNAs targeting the FSP1 (gene number: NM_002961), according to siRNA design principle, was synthesized by chemical synthesis and optimized by high‐performance liquid purification and demethylation. The sequence is as follows: 5′‐UGA ACA AGA CAG AGC UCA Att‐3′ (justice chain) and 5′‐UUG AGC UCU GUC UUG UUC ATT‐3′ (antisense chain). At the same time, control siRNA was synthesized chemically.

### 2.3 Cell culture

Mice were weighed and anaesthetized with a 1% pentobarbital sodium (10 mg/kg) through an intraperitoneal injection. When the mice were anesthetized, the chest was opened and the thoracic aorta was dissociated following the exit plane of the aorta to the entrance of the diaphragm. The outer membrane tissue was removed with forceps and cut into small pieces about 1 × 1 × 1 mm^3^. Every piece of tissue was evenly placed into a petri dish containing 10% foetal bovine serum at an interval of about 0.5 cm. Incubated at 37°C in humidified air with 5% CO_2_ for 96 hours, a small number of cells could be seen swimming out and sticking to the dish wall around the tissue block. The culture medium was replaced three times a week, and the passage was carried out when the cells reached 80%‐90% confluence about 7 days. The primary cells from the fourth to the eighth generations were used. After the AF purity was identified, the optimal concentration of FSP1 and siRNA‐FSP1 on AF was screened according to proliferation. In this study, the transfection or stimulation with stimulant was performed when cells reached 60%‐70% confluence. The transfection concentration of stimulant was as follows: 40 nmol/L for siRNA‐FSP1, 40 nmol/L for siControl, 40 nmol/L for FSP1, 20 μmol/L for AG490, 20 μmol/L for DKK, 20 μmol/L for FPS‐ZM1 and 20 μmol/L for Stattic.

### 2.4 Transfection

Cultured cells were transfected with siRNA‐FSP1 and lipofectamine 3000 (Invitrogen) following the manufacturer's protocol. The cells were seeded in a 6‐well plate and cultured to 30%‐40% confluence for cell transfection. Three 1.5‐mL EP tubes were prepared for liposome preparation: The first tube is for 125 μL opti‐MEM + 10 μL siControl (40 nmol/L); the second one is added with 125 μL opti‐MEM + 10 μL siRNA‐FSP1 (40 nmol/L); and the third one is filled with 250 μL opti‐MEM + 15 μL lipofectamine 3000. The liquid in the third tube (with lipofectamine 3000) was equally divided into the first and second tubes. Then, the liquid in the first and second tubes was incubated at 37°C for 15 minutes. The cultured cells in the 6‐well plate were respectively transfected with the liquid in the first and second tubes and incubated for 4‐6 hours in an incubator. And then, the culture liquid was substituted with the complete medium. The cells were cultured for an additional 48 hours and harvested at 48 hours when they reached 60%‐70% confluence.

### 2.5 Experimental cell grouping

AFs in the experiment were grouped into AF (untreated), siControl (negative siRNA duplex for FSP1), siRNA‐FSP1, FSP1 + siRNA‐FSP1, FSP1, FSP1 + AG490 (JAK/STAT specific blocker), FSP1 + DKK (Wnt specific blocker), FSP1 + FPS‐ZM1 (FSP1 specific blocker) and FSP1 + Stattic (JAK/STAT specific blocker).

### 2.6 Cell vitality

After intervention for 48 hours, MTT, EdU and adhesion assays were, respectively, performed. Migration experiment was observed at 0 hour, 12 hours and 24 hours, respectively, after treatment of stimulation.

#### 2.6.1 MTT

Cells were seeded in a 96‐well plate (1.5 × 10^4^ cells per well) and cultured overnight. Cell transfection or stimulation was performed when AF cells reached 60%‐70% confluence. After stimulation for 48 hours, the cell culture medium was replaced with 100 uL fresh culture medium containing 20 μL of MTT (5 mg/mL). After incubation at 37°C for 4 hours, 150 μL of dimethyl sulfoxide (1.1 g/mL) was added into the medium. Finally, after incubation for 1 hour at 37°C, absorbance at 490 nm of each well was measured by enzyme‐linked immunosorbent assay on a Bio‐Tek 311 Microtiter Plate Reader (Elx 800, Bio‐Tek). The optical density (OD) values were measured.

#### 2.6.2 EdU

Cultivated an appropriate number of cells in 6‐well plates (2.0 × 10^4^ cells per well) and performed the required stimulation for 48 hours. The pre‐heated EdU (20 μmol/L) working fluid (37ºC) was added to the 6‐well plates with an equal volume and continued to culture cells for 2 hours. After the completion of the EdU‐labelled cells, removed the culture solution and added 1 mL 4% polyformaldehyde, fixed at room temperature for 15 minutes. Removed the 4% polyformaldehyde and washed the cells three times with 1 mL PBS with 3% BSA per hole for 3‐5 minutes each time. Removed the PBS and incubated at room temperature for 10‐15 minutes with 1 mL PBS with 0.3% Triton per hole. Removed the permeable liquid and washed the cells 1‐2 times with 1 mL PBS with 3% BSA per hole for 3‐5 minutes each time. Removed the PBS with 3% BSA and added the Click reaction liquid incubating for 30 minutes at room temperature avoided light. Removed the Click reaction liquid and washed three times for 3‐5 minutes each time. After removing the washing liquid, added 1X Hoechst 33342 solution (1:1000 v/v) diluted with PBS 1 mL per hole and incubated for 10 minutes at room temperature avoided light. Removed 1X Hoechst 33342 solution and washed three times for 3‐5 minutes each time. Fluorescence could then be detected.

#### 2.6.3 Adhesion assay

Each well of the 96‐well plate was coated with fibronectin (10 mg/L) 200 μL and laminin (50 mg/L) 40 μL (Sigma), and dried at room temperature. Then, 1% bovine serum albumin was added into each well and incubated in a 37°C incubator for 1 hour. The cells were washed three times with PBS, trypsinized, harvested and adjusted to 8 × 10^5^ cells/mL with serum‐free DMEM. Then, 0.1 mL of cells were added into each well, transfected with stimulus for 20 hours, incubated with MTT (5 mg/mL) for 4 hours and supplemented with 200 μL DMSO (1.1 g/mL) after discarding supernatant. The OD of cells in each well was quantitated at 490 nm. Cell adhesion ratio was the adherent cell OD to total OD × 100%.

#### 2.6.4 Wound healing test

Cells were seeded in a 6‐well plate (5 × 10^4^ cells per well) and cultured overnight until 90%‐100% confluent. It is ensured that the six‐well plates were full of AF cells. Along the culture plate diameter, cells were scraped off with a sterilized self‐made scraper, which left a 1 mm width of scratch. Washed the cells with PBS three times, removed the assigned cells and added a serum‐free medium. Added stimulation and cultured in 37°C with 5% CO_2_ culture box. Wound healing area was observed, photographed and calculated by an image‐processing tool called Image J (Imagej v1.8.0, National Institutes of Health, America) in 0, 12 and 24 hours, respectively.

### 2.7 Flow cytometry

#### 2.7.1 Cell apoptosis

After 48 hours' stimulation, collected and suspended AF cells with PBS and counted the number of cells. Took 100 000 suspended cells, discarded the upper PBS after centrifugating and added 195 μL Annexin V‐FITC combing liquid to suspend cells. Added 5 μL Annexin V‐FITC and mixed gently. Added 10 μL propidium iodide (PI) staining solution and mixed gently. Incubated for 10‐20 minutes at room temperature (20‐25°C) away from light and then placed in an ice bathing. Lastly, cell apoptosis was analysed by flow cytometry. Annexin V‐FITC is green fluorescence and PI is red fluorescence.

#### 2.7.2 Cell cycle

After 48 hours' stimulation, cultured AF cells (1 × 10^5^ cells per well) were digested by trypsin and collected after centrifugating at 1500 rounds/min. The cells were washed two times with phosphate buffer solution (PBS), fixed with 75% ethanol at 4°C overnight and washed with PBS, and then, 100 uL RNase A was added to the cells. Finally, 400 uL PI was added to the AF cells away from light for 20 minutes, and then, cell cycle was analysed by flow cytometry.

### 2.8 Western blotting

After treatment of 48 hours with stimulation, the total protein was extracted and detected by Western blotting. The expression of cytokine protein was demonstrated by the ratio of integral optical density (IOD) between cytokine and β‐actin. AF cells were washed three times with cold PBS, thoroughly drained remanent PBS, extracted with RIPA and PMSF (100:1) for 30 minutes, scraped off the plate with a cold plastic cell scraper and then transferred into cold EP tube. These cells were sonicated for 15 seconds and centrifuged at 4°C for 15 minutes at 12 000*g*. The supernatant was collected and added loading buffer at 5:1 ratio. The mixture was heated at 95°C for 10 minutes. Finally, the protein and loading buffer mixture were stored at −20°C. Equal protein was separated on SDS‐PAGE and transferred to PVDF membranes, blocked with TBST containing 5% skim milk for 1 hour at room temperature, and washed three times for 5 minutes with TBST and then incubated overnight with first antibodies at 4°C. The membranes were probed overnight at 4°C with the following antibodies: p‐JAK2 (1:500; rabbit; Cell Signaling Technology; 125kD), JAK2 (1:1000; rabbit; Cell Signaling Technology; 120kD), p‐STAT3 (1:1000; rabbit; Cell Signaling Technology; 86kD), STAT3 (1:1000; rabbit; Cell Signaling Technology; 75kD), Wnt3a (1:500; rabbit; Cell Signaling Technology; 42kD), p‐β‐catenin (1:500; mouse; Cell Signaling Technology; 94kD), β‐catenin (1:1000; mouse; Cell Signaling Technology; 92kD), FSP1 (1:1000; rabbit; Cell Signaling Technology; 12kD), LC3B (1:1000; rabbit; Cell Signaling Technology; 14,16kD), RAGE (1:1000; rabbit; Abcam; 45kD), p62 (1:1000; rabbit; Abcam; 62kD), beclin‐1 (1:1000; rabbit; Abcam; 52kD), Apg7 (1:1000; rabbit; Abcam; 77kD), ICAM‐1 (1:1000; rabbit; Zhong Shan‐Golden Bridge Biological Technology; 110kD), VCAM‐1(1:1000; rabbit; Zhong Shan‐Golden Bridge Biological Technology; 85kD), MCP‐1(1:1000; rabbit; Zhong Shan‐Golden Bridge Biological Technology; 25kD), P4HA1 (1:1000; rabbit; Abcam; 61kD), TCF4 (1:1000; rabbit; Abcam; 71kD), collagen I (1:1000; mouse; Abcam; 130kD), collagen III (1:1000; rabbit; Abcam; 138kD) and β‐actin (1:2000; mouse; Zhong Shan‐Golden Bridge Biological Technology; 43kD). The membranes were washed and treated with corresponding horseradish peroxidase (HRP)‐conjugated secondary anti‐rabbit/mouse/IgG (1:5000) for 2 hours at room temperature. Then, the PVDF membranes were washed three times for 10 minutes. The membranes were briefly incubated with ECL detection reagent and then detected by using FluorChem Q System (ProteinSimple).

### 2.9 Reverse transcription quantitative PCR

Total cellular RNA was extracted from cultured AF cells after stimulation using TRIzol reagent (Invitrogen) according to the manufacturer's protocol. We synthesized complementary DNA using a Prime Script RT Master Mix Kit (Takara). Quantitative real‐time PCR was performed in duplicate with a SYBR Premix Ex TaqTM Kit (Takara). Primers for RT‐PCR are as follows: β‐actin (NM_007393.3; 5′‐GTGACGTTGACATCCGTAAAGA‐3′, 5′‐GTAACAGTCCGCCGCCTAGAAGCAC‐3′), FSP1 (NM_011311.2; 5′‐CCTGGGGAAAAGGACAGATGAA‐3′, 5′‐CATGGCAATGCAGGACAGGA‐3′), JAK2 (NM_001048177.2; 5′‐TGGAGTGGCTAAGCAGTTGGC‐3′, 5′‐TCAGGGGCTTATCTCCTCCAC‐3′), RAGE (NM_001271422.1; 5′‐TCCCGATGGCAAAGAAACACT‐3′, 5′‐GCAGGAGAAGGTAGGATGGGT‐3′), STAT3 (NM_011486.5; 5′‐GCTGACCAATAACCCCAAGAAC‐3′, 5′‐TGACACCCTGAGTAGTTCACACC‐3′), Wnt3a (NM_009522.2; 5′‐ATCTGGTGGTCCTTGGCTGTG‐3′, 5′‐ACTCCTGGATGCCCGCTTT‐3′) and β‐catenin (NM_001165902.1; 5′‐TTGCGGGAACAGGGTGCTAT‐3′, 5′‐ACGCCCTCCACAAACTGCT‐3′). The quantity of RNA used to synthesize cDNA was 1 μg. The PCR thermocycler conditions were as follows: denaturation at 95°C for 10 minutes, followed by 40 cycles at 95°C for 15 seconds and 60°C for 60 seconds. The data were analysed using the 2^−ΔΔCt^ method and normalized against β‐actin expression.

### 2.10 Immunocytochemistry

Cells in the six‐well plates were stimulated for 48 hours. After stimulation, the cells were washed with PBS three times for 5 minutes each time, fixed in 4% paraformaldehyde for 15 minutes, washed three times for 5 minutes each time, permeabilized with 0.1% Triton X‐100 for 20 minutes, blocked in normal goat serum for 30 minutes at room temperature and then incubated overnight with primary antibodies at 4°C in black humidified box. The primary antibodies are as follows: FSP1 (1:50; rabbit; Cell Signaling Technology; 12kD), RAGE (1:100; rabbit; Abcam; 45kD), Wnt3a (1:50; rabbit; Cell Signaling Technology; 42kD), p‐β‐catenin (1:50; mouse; Cell Signaling Technology; 94kD), p‐JAK2 (1:100; rabbit; Cell Signaling Technology; 125kD), p‐STAT3 (1:100; rabbit; Cell Signaling Technology; 86kD), LC3B (1:100; rabbit; Cell Signaling Technology; 14,16kD), P4HA1 (1:100; rabbit; Abcam; 61kD), β‐catenin (1:50; mouse; Cell Signaling Technology; 94kD) and TCF4 (1:100; rabbit; Abcam; 71kD). On the second day, these cells were washed, incubated with appropriate rhodamine (TRITC)‐conjugated secondary antibody (goat antimouse/rabbit antibody; 1:100) and FITC‐conjugated secondary antibody (goat antimouse/rabbit antibody; 1:100) for 1 hour and then counterstained nucleus with DAPI for 5 minutes. The slides were washed in PBST three times for 5 minutes each time, mounted with fluorescent mounting medium and then observed the image under a fluorescence microscope.

### 2.11 Transmission electron microscope (TEM) analysis

Immobilization of materials: After 48 hours' stimulation, AFs were discarded the culture liquid, added the electron microscope fixation liquid at 4°C for 2‐4 hours, centrifuged at low speed, wrapped with 1% agarose and then rinsed three times with 0.1 mmol/L phosphate buffer (PH 7.4), 15 minutes every time. Post‐fixation: fixed with 1% osmic acid and 0.1 mmol/L phosphate buffer (PH7.4) mixture at room temperature (20°C) for 2 hours and rinsed with 0.1 mmol/L phosphate buffer (PH7.4) three times, 15 minutes every time. Dehydration: The cell samples were dehydrated with different concentrations of alcohol and acetone. Permeation: The samples were placed in a mixture of acetone and embedding agent (1:1) for 2 hours, infiltrated overnight in a mixture of acetone and embedding agent (2:1), and then placed in pure embedding agent for 6 hours. Lastly, pure embedding medium was poured into the embedding board, and the cell samples were inserted into embedding plate overnight in 37°C oven. Then, cell samples were placed into 60°C oven for 48 hours. Slicing: Ultrathin microtome was used to cut cell samples into 60‐80 nm sections. Staining: slices were stained by 2% uranium acetate saturated alcohol solution and lead citrate for 15 minutes respectively and dried overnight at room temperature. Then, we observed under a transmission electron microscope to collect image analysis.

### 2.12 Statistical Analysis

Results were expressed as the mean ± SD and analysed using GraphPad Prism software. All experiments presented within this study were reflective of at least three replicate studies with similar results. The comparison among multiple groups was conducted by one‐way anova. The comparison between two groups was performed by *t* test. Ratio comparison involved chi‐square analysis. A *P*‐value of < 0.05 was considered to be statistically significant.

## 3 RESULTS

### 3.1 AF Identification and the optimal stimulation concentration

**Figure 1:**
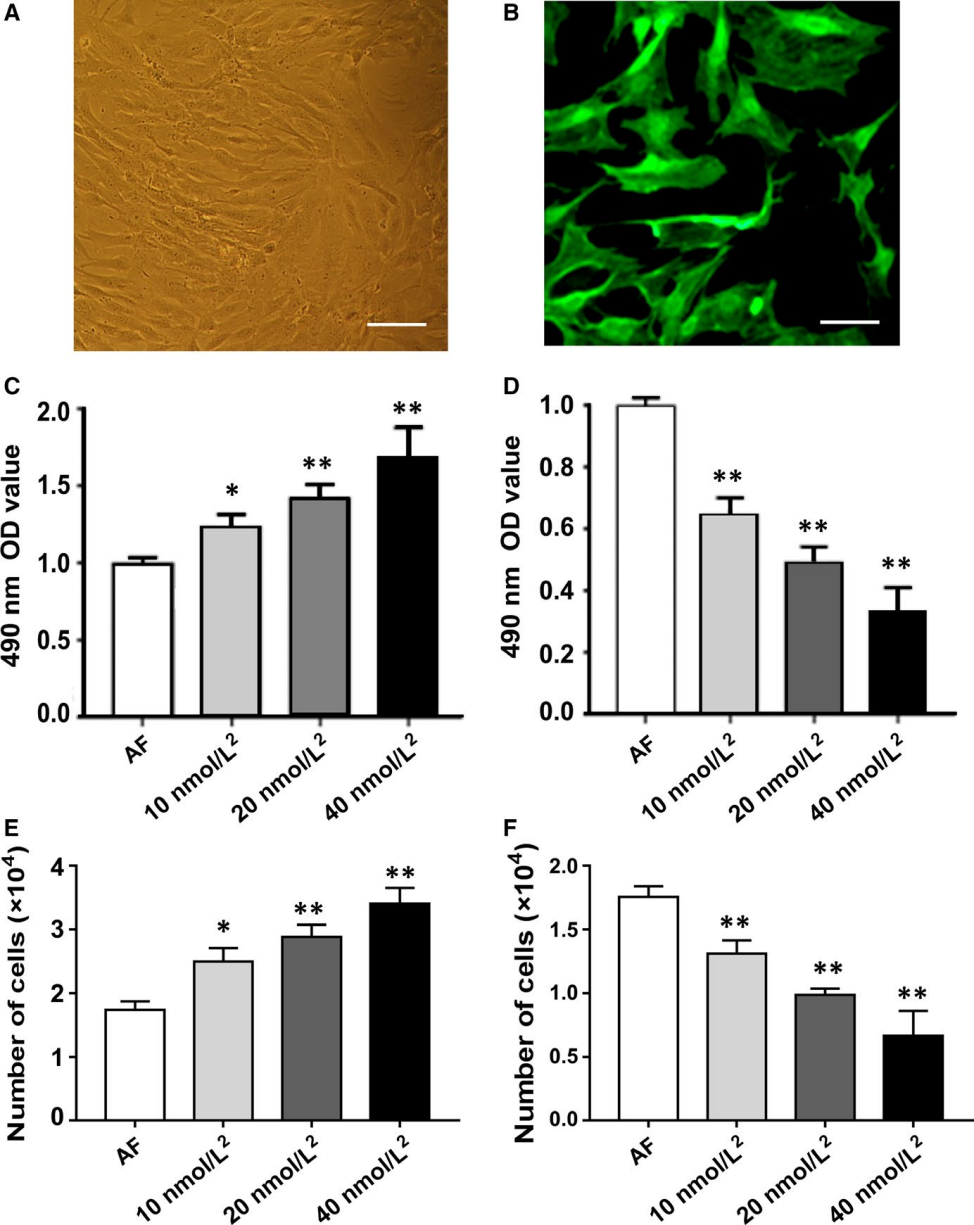
Identification of AF cells and screening for optimum stimulating concentration of FSP1. A, Cultured AF cells before immunofluorescence staining in bright field. Scale bar represents 50 μm. B, Antivimentin (+) cells with fluoresceinisothiocyanate (FITC) fluorescence in dark field. Scale bar represents 20 μm. C, Effects of different concentrations of FSP1 on AF cell proliferation. D, Effects of different concentrations of siRNA‐FSP1 on AF cell proliferation. E, The number of proliferative AF cells was showed after different concentrations of FSP1 stimulation for 48 h. F, The number of proliferative AF cells was showed after different concentrations of siRNA‐FSP1 stimulation for 48 hours. (Data presented as mean ± SD; the comparison of multiple groups was performed by anova; versus AF group, *P < 0.05, **P < 0.01)

All cultured cells demonstrated only negative staining for α‐actin and only positive staining for vimentin, which suggested 100% purity of cultured AFs (Figure 1A,B). In addition to vimentin expression in AFs, FSP1 and P4HA1 were not only fundamentally expressed but also highly expressed with FSP1 stimulation in AF cells (Figure S1). MTT assay displayed that the OD value of AFs (Figure 1C) and the number of proliferative cells (Figure 1E) gradually increased with the increase in FSP1 concentration, indicating that the proliferative activity of AFs gradually increased. On the contrary, as the concentration of siRNA‐FSP1 increased, the OD value of AFs (Figure 1D) and the number of proliferative cells (Figure 1F) gradually decreased, indicating that FSP1 knockdown could significantly reduce the reproductive capacity of AFs. According to the effect of FSP1 and siRNA‐FSP1 on cell proliferation, finally, 40 nmol/L of FSP1 or siRNA‐FSP1 was selected as the optimal stimulation concentration. (Figure 1C,D).

### 3.2 Effects of FSP1 and siRNA‐FSP1 on AF vitality

**Figure 2:**
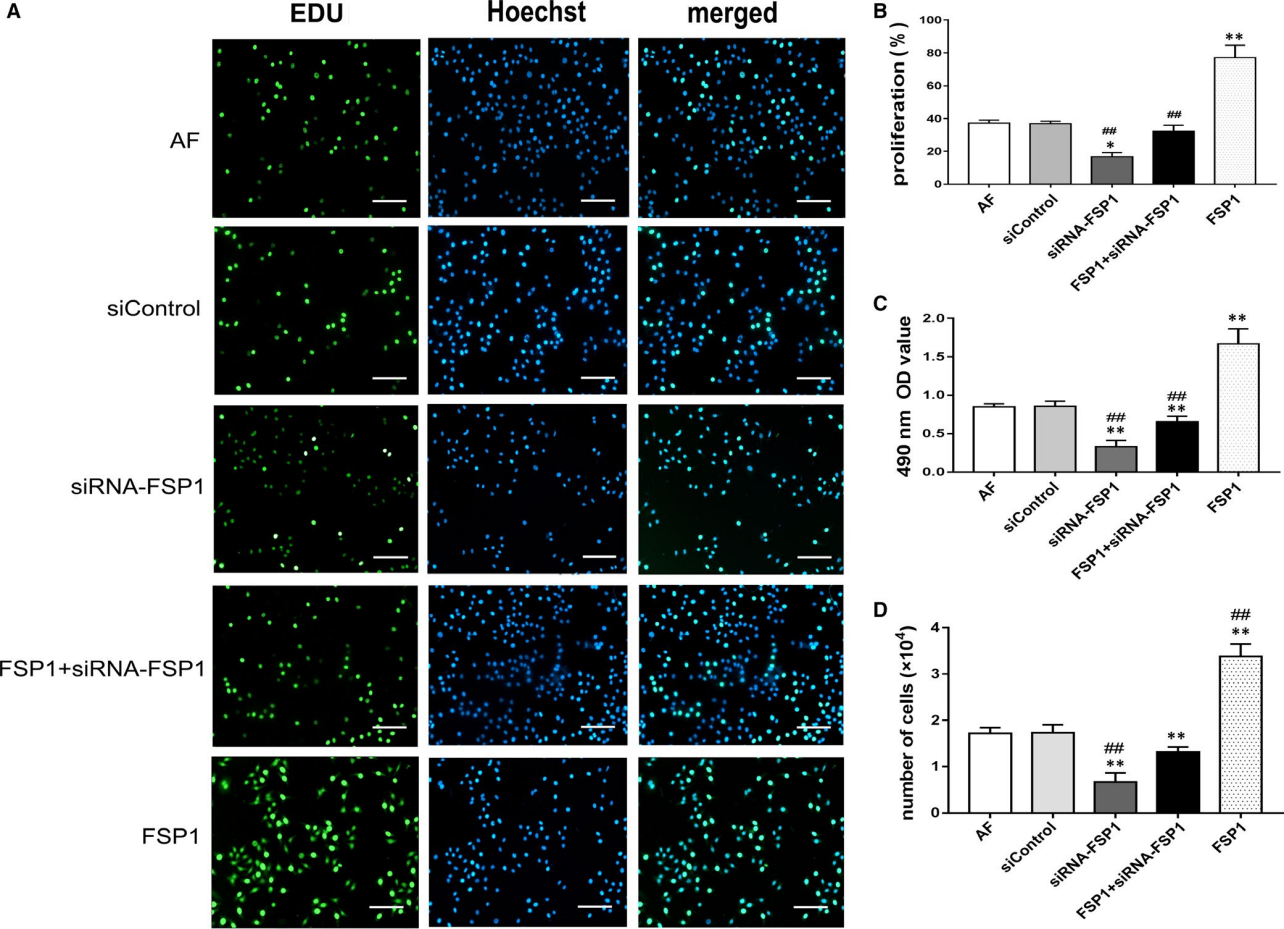
FSP1 promotes AF cell proliferation. After 48 h stimulation with 40 nmol/L FSP1, cell proliferative assays were evaluated. A, EdU incorporation assay (×20). Blue, Hoechst labelling of cell nuclei; green, EdU labelling of nuclei of proliferative cells. Scale bars represent 100 μm. B, Bar graph demonstrated the comparison of proliferative ratio among different groups. The more the green‐stained nuclei, the stronger the AF cells proliferation. C, Bar graph showed the comparison of 480 nm OD values in different groups. MTT assays indicated that FSP1 promoted AF cells proliferation; siRNA‐FSP1 inhibited AF cells proliferation. D, The number of proliferative AF cells was showed after treatment for 48 h. (Data presented as mean ± SD; the comparison of multiple groups was performed by anova; vs AF group, *P < 0.05, **P < 0.01; vs FSP1 group, #*P* < 0.05, ##*P* < 0.01)

**Figure 3:**
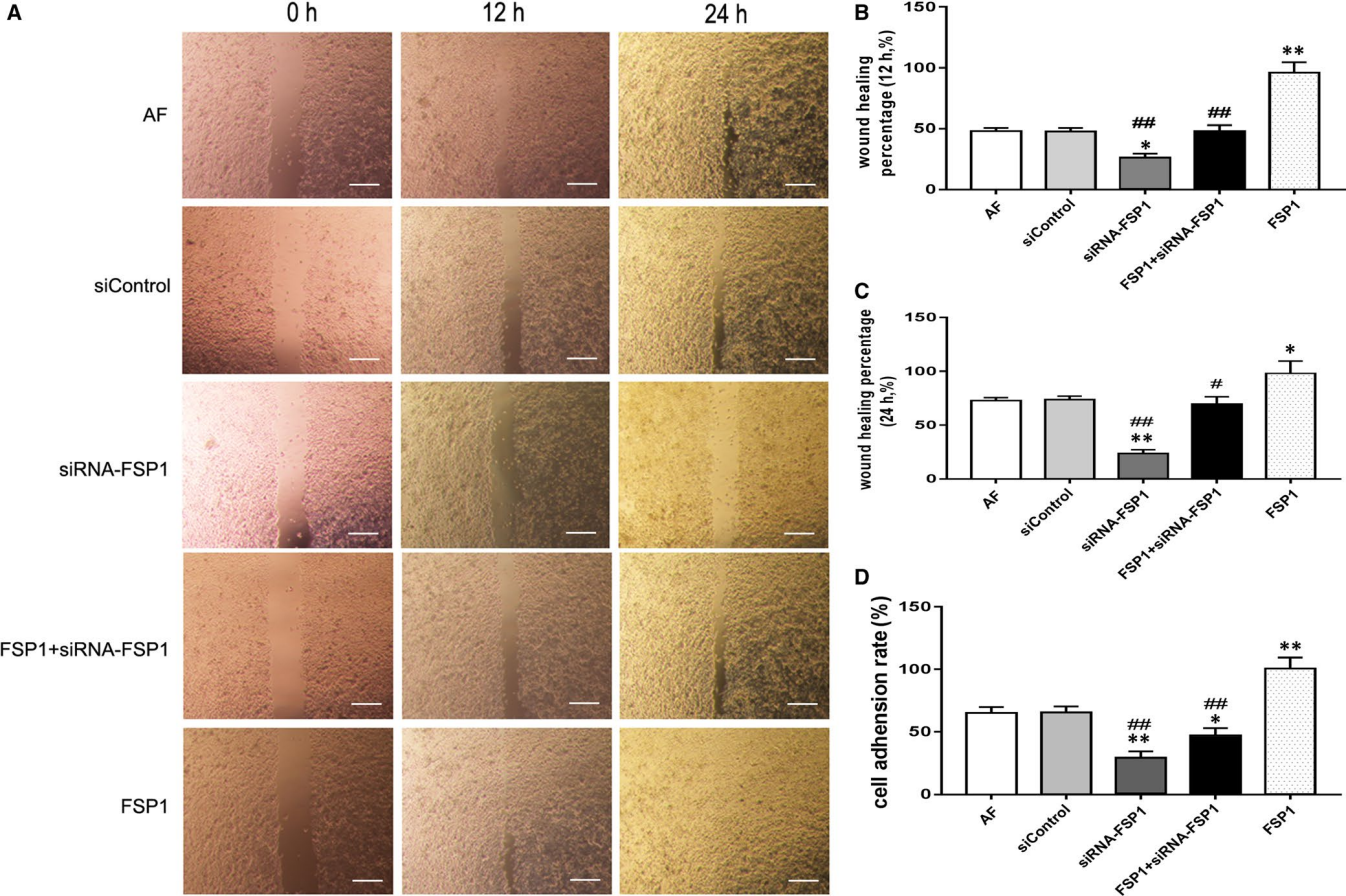
FSP1 promotes cell migration and adhesion. A, Wound healing test was observed at 12 h and 24 h, respectively, after 40 nmol/L FSP1 stimulation. Scale bars represent 200 μm. Wound healing area was calculated by Image J. B, The comparison of 12 h' wound healing percentage. C, The comparison of 24 h' wound healing percentage. D, Cell adherent ability was calculated after 40 nmol/L FSP1 stimulation for 24 h. (Data presented as mean ± SD; the comparison of multiple groups was performed by anova; vs AF group, *P < 0.05, **P < 0.01; vs FSP1 group, #*P* < 0.05, ##*P* < 0.01)

AF proliferative activity was calculated by Edu‐labelled AF counting and MTT assay (Figure 2). The proliferative ratio and OD were significantly higher in the FSP1 group than those in the other groups. The group of siRNA‐FSP1 had a lower proliferative ratio and OD than those in AF, siControl and FSP1 groups. Compared with FSP1 group, the intervention groups had dramatically decreased proliferation ratio and OD values of AF cells after treatment with signalling pathway blockers (AG490, DKK, FPS‐ZM1 and Stattic; Figure S2). By comparison, the values of wound healing percentage and adhesion ratio were significantly higher in the FSP1 group than those in other groups. These values were lower in the siRNA‐FSP1 group than those in AF, siControl and FSP1 groups (Figure 3). Compared with FSP1 group, the wound healing percentage and adhesion ratio in intervention groups treated with signalling pathway blockers (AG490, DKK, FPS‐ZM1 and Stattic) were also reduced (Figure S3). Therefore, FSP1 could effectively stimulated AF cells proliferation, migration and adhesion. Contrarily, siRNA‐FSP1 counteracted the effects of FSP1 on AFs.

### 3.3 Effects of FSP1 and siRNA‐FSP1 on AF cell cycle and apoptosis

**Figure 4:**
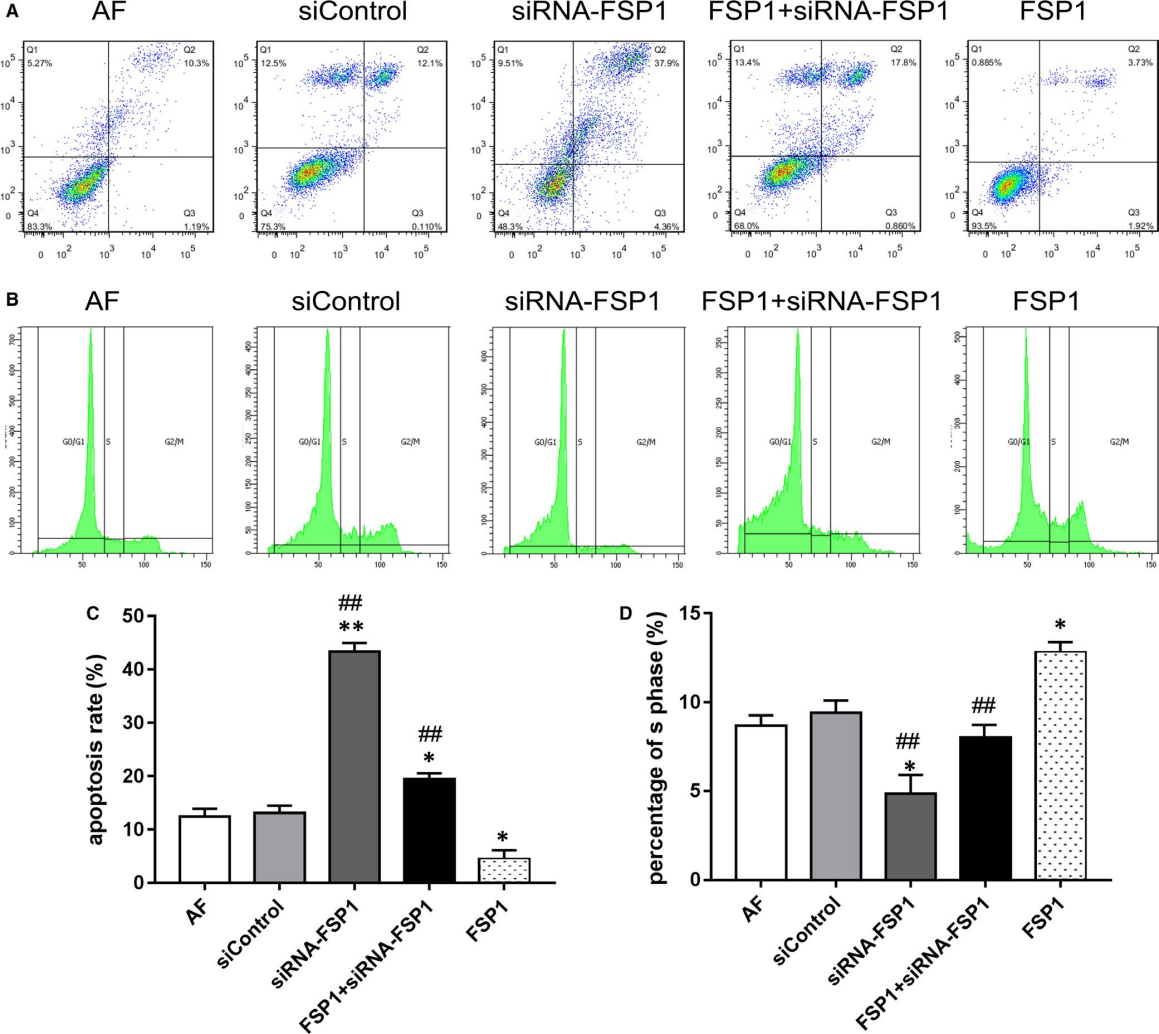
FSP1 increases AFs number in S phase and decreases cellular apoptosis. A, Flow cytometry measured the cell apoptosis after 40 nmol/L FSP1 stimulation for 48 h. AF cells apoptosis was detected by Annexin V/PI double staining. B, Flow cytometry measured the cell cycle after 40 nmol/L FSP1 stimulation for 48 h. Cell cycle distribution was measured by flow cytometry using propidium iodide stain. The percentage of S phase of AF cells with different treatment was shown in the representative data. C, Bar graph demonstrated comparison of apoptosis ratio. D, Bar graph demonstrated comparison of S phase in different groups. (Data presented as mean ± SD; the comparison of multiple groups was performed by anova; vs AF group, *P < 0.05, **P < 0.01; vs FSP1 group, #*P* < 0.05, ##*P* < 0.01)

The FSP1 group showed a trend of decreased AF number in apoptotic phase (Figure 4A) and a significant increase in AF number in S phase compared with other groups (Figure 4B). Contrarily, compared with AF, siControl and FSP1 groups, the siRNA‐FSP1 group showed the increased number of apoptosis(Figure 4A) and the decreased number of S phase (Figure 4B). The apoptosis rate was increased in the signal pathway blocker groups (FSP1 + AG490, FSP1 + DKK, FSP1 + FPS‐ZM1 and FSP1 + Stattic) and a decreased number of AFs in the S phase compared with FSP1 group (Figure S4). Thus, FSP1 could reduce the apoptosis of AF cells and promote the proportion of cells in S phase.

### 3.4 Effects of FSP1 and siRNA‐FSP1 on the expression of cytokines in RAGE, JAK2/ STAT3 and Wnt/β‐catenin pathways and some inflammatory factors

**Figure 5:**
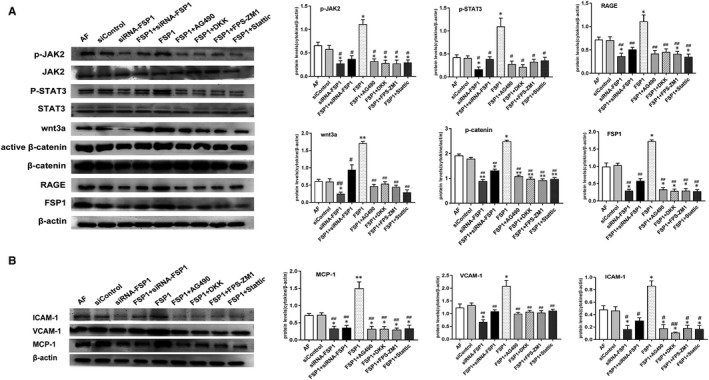
FSP1 up‐regulates the expression of molecules in RAGE, JAK2/STAT3 and Wnt3a/β‐catenin signaling pathways and related proinflammatory cytokines. A, After 48 h' intervention, protein expression of related cytokines in RAGE, wnt/β‐catenin and JAK2/STAT3 pathways. When added FSP1(40 nmol/L) stimulation, the signaling pathway proteins were increased. Contrary, siRNA‐FSP1 (40 nmol/L) and signaling pathway blockers (AG490:20 μmol/L, DKK:20 μmol/L, FPS‐ZM1:20 μmol/L and Stattic: 20 μmol/L) reduced the expression of signaling pathway proteins. Bar graph represented the comparison of proteins expression in different groups. B, Western blotting detected the expression of MCP‐1, VCAM‐1 and ICAM‐1. AF cells were treated for 48 h. MCP‐1, VCAM‐1 and ICAM‐1 levels increased after the stimulation of 40 nmol/L FSP1 and decreased after the stimulation of 40 nmol/L siRNA‐FSP1 and signaling pathway blockers (AG490:20 μmol/L, DKK:20 μmol/L, FPS‐ZM1:20 μmol/L and Stattic: 20 μmol/L). Bar graph represents the comparison of proteins expression in different groups. Values were normalized using β‐actin protein as control. (Data presented as mean ± SD; the comparison of multiple groups was performed by anova; vs AF group, *P < 0.05, **P < 0.01; vs FSP1 group, #*P* < 0.05, ##*P* < 0.01)

When treated with FSP1 stimulation, the expression of RAGE, JAK2/STAT3 and Wnt/β‐catenin was higher in FSP1 group than those in other groups, whereas the siRNA‐FSP1 reduced the expression of RAGE, JAK2/STAT3 and Wnt/β‐catenin. When the blockers (AG490, DKK, FPS‐ZM1 and Stattic) for signalling pathway were added, the expression of these signalling molecules was also decreased compared with the FSP1 group. Similarly, compared with other groups, FSP1 group had higher expression of some inflammatory factors (MCP‐1, VCAM‐1 and ICAM‐1) and siRNA‐FSP1 group had lower expression of these inflammation cytokines (Figure 5). The expression of these inflammatory factors in groups with blockers (AG490, DKK, FPS‐ZM1 and Stattic) was inhibited compared with FSP1 group. Thus, we speculate that FSP1 could promote not only the expression of these pathway proteins but also the expression of these inflammatory factors.

### 3.5 Expression of mRNA of Wnt3a, β‐catenin, RAGE, FSP1, JAK2 and STAT3

**Figure 6:**
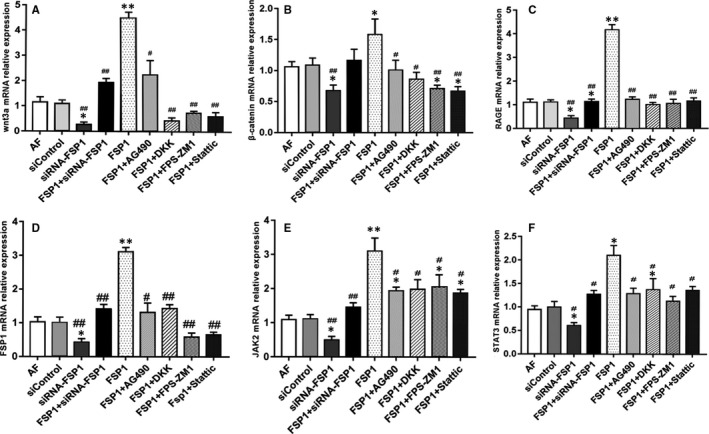
The enhanced expression of mRNA of RAGE, FSP1, JAK2/STAT3 and Wnt3a/β‐catenin after stimulation of FSP1 for 48 h. The expression of mRNA of wnt3a, β‐catenin, RAGE, FSP1, JAK2 and STAT3 was tested by RT‐PCR. These histograms illustrated the contrast of expressions. FSP1 up‐regulated the mRNA expression of signalling pathway molecules. Contrarily, siRNA‐FSP1 and signalling pathway blockers deregulated the mRNA of signalling pathway molecules. (Data presented as mean ± SD; the comparison of multiple groups was performed by anova; vs AF group, *P < 0.05, **P < 0.01; vs FSP1 group, #*P* < 0.05, ##*P* < 0.01)

Compared with other groups, FSP1 group had higher level of mRNA expression of Wnt3a, β‐catenin, RAGE, FSP1, JAK2 and STAT3. In the contrary, siRNA‐FSP1 group had a decreased level of mRNA expression compared with AF, siControl and FSP1 groups (Figure 6). Thereby, the expression of these signalling proteins can be induced by FSP1 stimulation. Moreover, signalling pathway blockers (AG490, DKK, FPS‐ZM1 and Stattic) can inhibit the gene expression of Wnt3a, β‐catenin, RAGE, FSP1, JAK2 and STAT3.

### 3.6 The expression of FSP1, β‐catenin and TCF4 activated by Wnt3a

To investigate the potential relationship between Wnt3a and FSP1, we evaluated the expression levels of FSP1, β‐catenin and TCF4. Compared with AF group, both immunofluorescence assay and Western blotting showed the enhanced expression of FSP1, β‐catenin and TCF4 after Wnt3a (50 nmol/L) stimulation (Figure S5). The experiment demonstrates that FSP1 activation mediated by Wnt3a/β‐catenin might be correlated with TCF4.

### 3.7 Comparison of immunofluorescence of signalling pathway proteins

**Figure 7:**
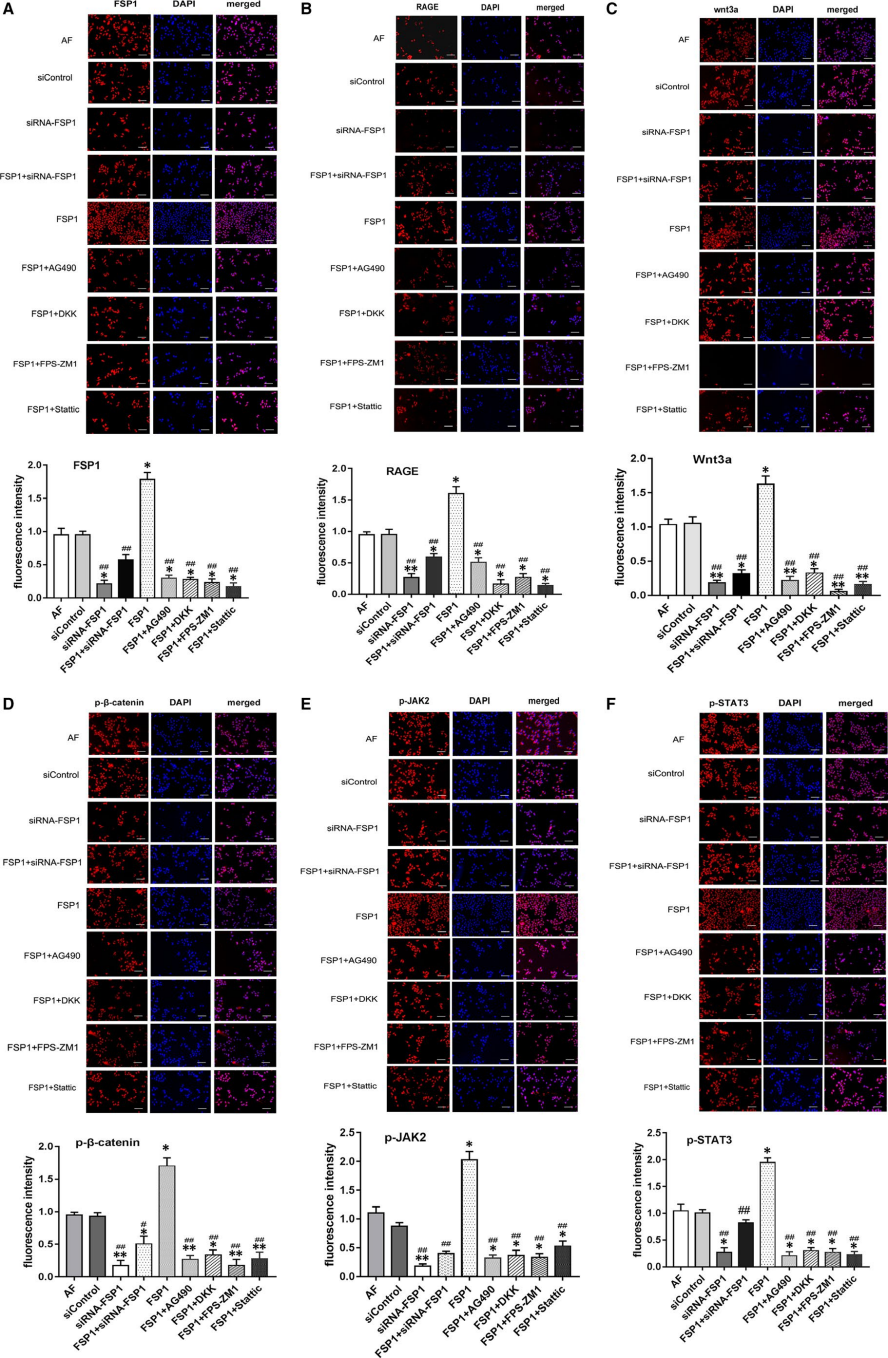
Immunofluorescence assays demonstrate that the expression of proteins in signalling pathways dramatically increased with FSP1 treatment. TRITC‐labelled protein expression of related cytokines in immunocytochemistry staining (×20). Immunocytochemistry demonstrated the expression of wnt3a, p‐β‐catenin, RAGE, FSP1, p‐JAK2 and p‐STAT3. AF cells were stimulated for 48 h and immunostained for these proteins. DAPI (blue) staining to visualize nuclei (magnification, ×200). Scale bars represent 100 μm. Graph showed quantitative analysis of immunofluorescent intensity for wnt3a, p‐β‐catenin, RAGE, FSP1, p‐JAK2 and p‐STAT3. (Data presented as mean ± SD; the comparison of multiple groups was performed by anova; vs AF group, *P < 0.05, **P < 0.01; vs FSP1 group, #*P* < 0.05, ##*P* < 0.01)

After 48 hours' stimulation, the fluorescence intensity of cell signalling pathway proteins in FSP1 group was stronger than those in other groups. The fluorescence density of signalling pathway blocker groups (FSP1 + AG490, FSP1 + DKK, FSP1 + FPS‐ZM1 and FSP1 + Stattic) was significantly lower than FSP1 group. Compared with AF, siControl and FSP1 groups, siRNA‐FSP1 group had weaker fluorescence intensity. Thus, FSP1 can enhance the expression of signalling pathway proteins. Contrary, siRNA‐FSP1 can reduce the expression of these proteins. (Figure 7).

### 3.8 Autophagic detection

**Figure 8:**
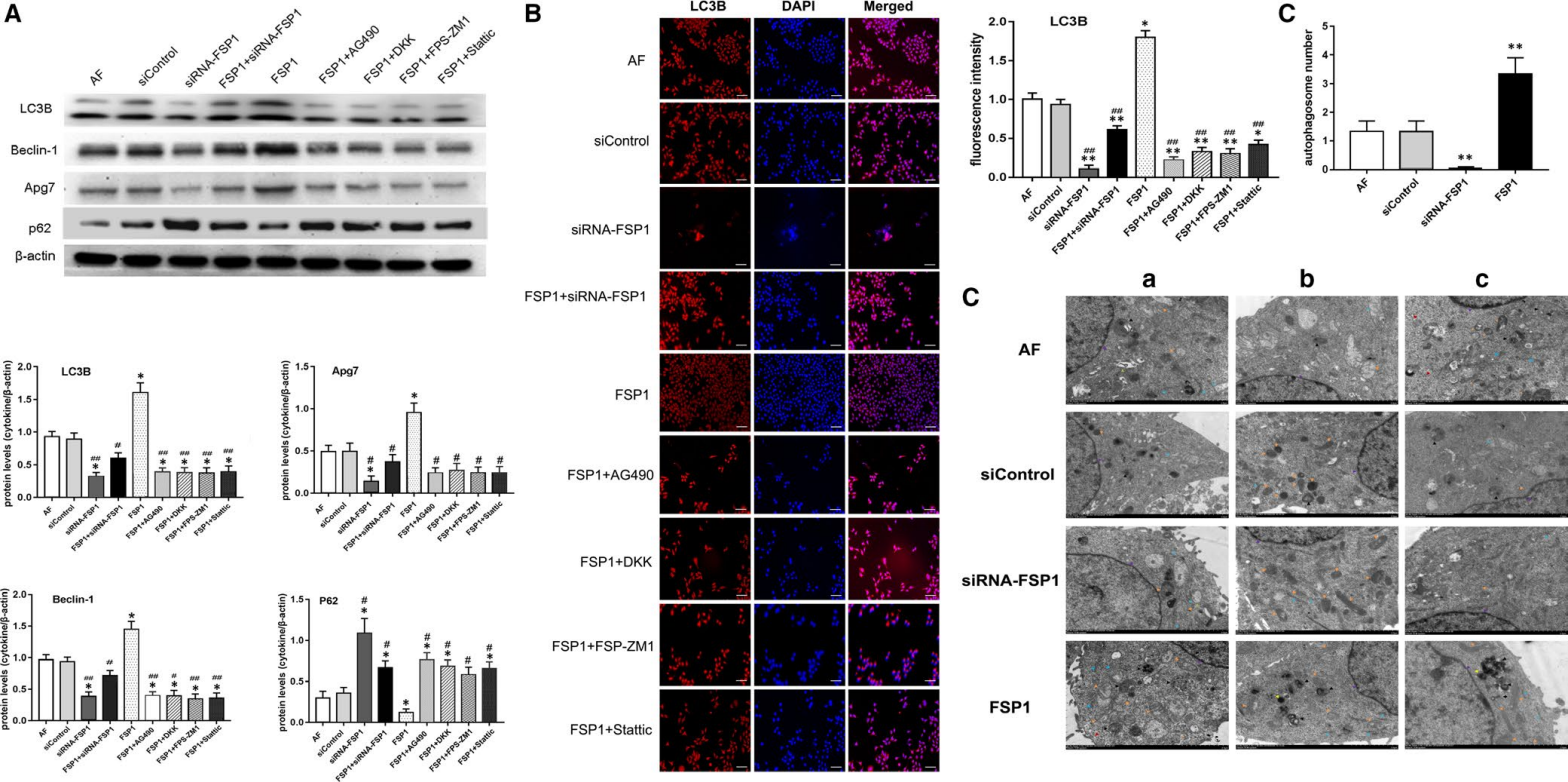
FSP1 promotes autophagy in AF cells. A, Autophagy‐related proteins (such as LC3B, beclin‐1, Apg7 and p62) were detected by Western blotting. B, The changes of LC3B in different groups were detected by immunofluorescence. Scale bars represent 100 μm. Graph showed quantitative analysis of immunofluorescent intensity for LC3B. C, Autophagosome was observed by TEM. For each group, we randomly selected 3 samples for observation. Representative images of autophagosome in AF cells in different groups were shown. The colour signs in the image represented as follows: black symbol (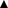): autophagy‐lysosome; purple symbol (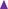): nucleus; orange symbol (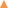): mitochondria; blue symbol (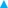): rough endoplasmic reticulum; yellow symbol (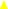): primary lysosome; green symbol (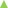): suspected mitochondrial swelling or vacuoles. The number of autophagosome was the highest in FSP1 (40 nmmol/L) group. Autophagosome was hardly seen in siRNA‐FSP1 (40 nmmol/L) group. The autophagy level of AF group was similar to siControl group, but it was lower than the FSP1 group and higher than the siRNA‐FSP1 group. (Data presented as mean ± SD; the comparison of multiple groups was performed by anova; vs AF group, *P < 0.05, **P < 0.01; vs FSP1 group, #*P* < 0.05, ##*P* < 0.01)

Western blotting showed that autophagy‐related proteins such as LC3B, beclin‐1 and Apg7 were higher in FSP1 group than those in other groups, and P62 was lower in FSP1 group than those in other groups. Compared with AF, siControl and FSP1 groups, siRNA‐FSP1 group had the decreased expression of LC3B, beclin‐1 and Apg7 and the increased expression of p62. When signalling pathway blockers were added, the expression of LC3B, beclin‐1 and Apg7 decreased and P62 increased compared with FSP1 group. (Figure 8A). The fluorescence intensity of LC3B in FSP1 group was stronger than that in other groups. The group of siRNA‐FSP1 had weaker fluorescence intensity than that in AF, siControl and FSP1 groups (Figure 8B). After stimulation of FSP1 for 48 hours, TEM images showed that the autophagy level increased compared with AF and siControl groups. Contrarily, after transfection of siRNA‐FSP1 for 48 hours, the level of autophagy was inhibited (Figure 8C). These results strongly suggest that exogenous FSP1 is an induction agent of autophagy in AF cells. Western blotting demonstrated that the expression levels of LC3B, collagen I and collagen III were higher in FSP1 group compared with AF and siRNA‐FSP1 groups. (Figure S6). The result indicates that autophagy induced by FSP1 promoted collagen deposition in AFs.

## 4 DISCUSSION

Prior to this study, no information existed on the functional consequences of increased or decreased FSP1 protein levels in regulation of AFs biofunctions. As previous research stated, FSP1 plays multiple roles role in cellular biofunctions through signalling transduction.^7^, ^14^ It is expressed in a variety of cells and highly expressed in fibrotic diseases of the heart, lung, liver, brain and kidney.^6^, ^15^ Our study displayed FSP1 could not only express but also be overexpressed in AFs (Figure 1C,D; Figure 2). In addition to the expression of FSP1 and vimentin proteins, prolyl‐4‐hydroxylase (P4H) also has basic expression under the condition of ordinary medium and higher expression after FSP1 stimulation in AF cells (Figure S1). P4HA1 is the major isoenzyme of P4H in most cells and essential for collagen synthesis, maturation and secretion.^19^, ^20^

Consistent with previous studies,^10^, ^14^ our data documented with EdU, MTT and migration assays showed that FSP1 overexpression significantly augmented cell proliferation, adhesion and migration abilities (Figure 1C,E; Figures 2, 3), alleviated cellular apoptosis and increased the cellular percentage in S phase (Figure 4). In contrast, FSP1 knocked down significantly reduced AFs proliferation, adhesion and migration ability (Figure 1D,F; Figures 2, 3), enhanced cellular apoptosis and decreased the cellular percentage in S phase (Figure 4). In our study, we demonstrated that FSP1 could promote AFs proliferation by affecting cell cycle, adhesion, apoptosis and migration (Figures 1, 2, 3, 4). To our knowledge, this was the first time we proved the effect of FSP1 on AFs biofunction including apoptosis (Figure 4).

As a well‐accepted interaction partner for RAGE, FSP1 coordinates the JAK/STAT signal transduction to activate molecular receptors in target cells.^21^ JAK/STAT pathway is involved in many important biological processes such as cell proliferation, differentiation, apoptosis, angiogenesis and immune regulation.^22^, ^23^ JAK is divided into four subtypes, and STAT has seven subtypes.^23^ Currently, JAK2/STAT3 has more evidence of cardiovascular biological effects.^22^, ^23^ Additionally, FSP1 is also the target gene for the Wnt/β‐catenin pathway.^11^ It is generally known that Wnt signalling is subdivided into canonical (β‐catenin‐dependent) and non‐canonical (β‐catenin‐independent) pathways. Of which, Wnt3a is one of the most highly studied canonical members.^24^ Wnt/β‐catenin can up‐regulate the expression of STAT3 and increase the cellular capacity of adhesion, migration and proliferation.^16^ Emerging evidence have showed that Wnt/β‐catenin signalling pathway plays an important role in regulating vascular cellular functions.^16^ However, so far it remains elusive that the modulatory mechanisms of RAGE, JAK/STAT3 and Wnt/β‐catenin signalling molecules for the cellular biofunctions (such as adhesion, proliferation, apoptosis and autophagy) in AFs. As demonstrated by our data, the mRNA expression and protein expression of RAGE, JAK2/STAT3 and Wnt3a/β‐catenin were, respectively, down‐regulated in AFs infected with siRNA targeting the FSP1 and were up‐regulated after FSP1 stimulation (Figures 5, 6, 7). Additionally, the expression of these signalling molecules, respectively, decreased as we separately added specific blockers (FPS‐ZM1 for RAGE; AG490 for JAK2; Stattic for STAT3; and DKK‐1 for Wnt) (Figures 5, 6, 7). Therefore, these results suggest the effect of FSP1 on AF cells through RAGE, JAK/STAT and Wnt/β‐catenin signalling pathways.

Apart from FSP1 activating FSP1‐RAGE‐STAT3 signalling axis to increases cell proliferation and resistance to apoptosis,^14^ as a RAGE agonist, FSP1‐RAGE‐Wnt pathway also mediates autophagy inhibition and promotes cell proliferation.^6^ In turn, Wnt/β‐catenin signalling can transcriptionally regulate STAT3 expression.^16^ In contrast, FSP1‐induced proliferation and migration can be inhibited with RAGE, JAK/STAT or/and Wnt/β‐catenin specific blockers.^14^ Similar to some results of previous study, in our study, FSP1 stimulation of AFs revealed that, as the expression of RAGE increased, the expression of JAK2, STAT3, Wnt3a and β‐catenin simultaneously increased (Figures 5, 6, 7). By contrast, after the intervention of siRNA‐FSP1 or specific inhibitors, as the expression of RAGE decreased, the expression of JAK2, STAT3, Wnt3a and β‐catenin also synchronously reduced (Figures 5, 6, 7), indicating that a crosstalk emerges among RAGE, JAK2/STAT3 and Wnt3a/β‐catenin pathways in the signal transduction of AF growth. Therefore, we could definitely assume that RAGE, JAK2/STAT3 and Wnt3a/β‐catenin pathways interact and jointly constitute a molecular network in regulating AF cytobiological functions.

It is worth mentioning that Wnt3a/β‐catenin had higher expression in FSP1 group than other groups in our study (Figures 5, 6, 7). The results indicate Wnt3a may directly interact with FSP1. So, we explored the possibility of interaction between Wnt3a and FSP1 in AF cells. Consistent with previous research results, the protein levels and fluorescence intensity of β‐catenin, FSP1 and TCF4 were increased in AFs stimulated by Wnt3a (Figure S5). It has been verified that FSP1 was the target of Wnt/β‐catenin signalling because FSP1 promoter contains a T‐cell factor (TCF) binding site that could be bound and activated by β‐catenin.^25^, ^26^ Conversely, after stimulation of FSP1, the expression of Wnt3a was also elevated (Figures 5, 6, 7). Thus, combined with the former study findings, we speculated that the TCF4 might be the mutual connector between Wnt3a and FSP1 in AFs.

As reported in our previous and other studies, fibroblast can secrete a wide array of cytokines, chemokines and inflammatory cytokines (such as MCP‐1, VCAM‐1 and ICAM‐1) in response to some stimuli,^27^, ^28^ amplifying the inflammatory response and contributing to cardiovascular remodelling.^27^, ^28^ It has been shown that the activation of RAGE, JAK/STAT and Wnt/β‐catenin signalling boosts a large number of inflammatory molecules, interacts with the proinflammatory cytokines and triggers cellular dysfunction in numerous pathophysiological processes.^16^, ^29^ During the early stage of the inflammatory response, fibroblasts may promote monocyte migration (through MCP‐1 secretion) and adhesion to the cellular surface (via ICAM‐1/VCAM‐1‐mediated binding).^27^ Conversely, MCP‐1 knockout produced inefficient cardiac remodelling, characterized by a prolonged inflammatory phase and a delay in the formation of granulation tissue and wound healing.^27^ Our study indicates FSP1 overexpression elevated the expression of MCP‐1, ICAM‐1 and VCAM‐1 accompanied by the overexpression of signalling molecules in RAGE, JAK/STAT and Wnt/β‐catenin pathways in the course of AFs growth (Figure 5). Inversely, FSP knockdown generated under‐expression of these inflammatory cytokines and signalling molecules (Figure 5). According to the literature, Wnt/β‐catenin may have both proinflammatory and anti‐inflammatory effects depending on the cell type, stimulus and cellular environment.^16^ In this work, it is obviously shown that Wnt3a/β‐catenin coordinated with RAGE and JAK2/STAT3 pathways to play a proinflammatory role in the process of cell proliferation mediated by FSP1 (Figure 5). Taken together, our findings represent an initial approach that RAGE, JAK2/STAT3 and Wnt3a/β‐catenin pathways coordinate to induce a proinflammatory cytokine profile characterized by high levels of MCP‐1, ICAM‐1 and VCAM‐1 (Figure 5).

Multiple evidence revealed that RAGE, JAK/STAT and Wnt/β‐catenin molecules are involved in multiple signalling pathways not only inflammation, proliferation and apoptosis, but also autophagy.^6^, ^11^, ^12^, ^13^, ^16^, ^30^ It is reported that the regulation of autophagy plays an important role in FSP1‐RAGE‐mediated cell survival.^6^, ^12^ Additionally, in a rat experimental model, the expression levels of p‐JAK2, p‐STAT3 and LC3B were significantly higher than those in the AG‐490 (inhibitor for JAK2) intervention group.^30^ Autophagy is a catabolic process in which cytoplasmic proteins or whole organelles are sequestrated and degraded by autolysosomes. Evidence from numerous studies show that autophagy is a double‐edged sword in cell biology, acting both as a cell growth suppressor and a survival protector.^6^, ^31^ Autophagy could promote cell survival, but excessive autophagy would contribute to cell injury and apoptosis.^12^, ^32^, ^33^ Unexpectedly, some study demonstrated FSP1 inhibited starvation‐induced autophagy to promote tumour cell viability via the Wnt/β‐catenin pathway in a RAGE‐dependent manner.^6^ Contrarily, the inhibitory effect of FSP1 on autophagy and its promotion role in cell proliferation was abolished using FPS‐ZM1 (inhibitor for RAGE).^6^ Mounting evidence has been reported to show the paradox role of autophagy in pro‐survival and pro‐apoptosis.^6^, ^12^ These conflicting results may be explained by differences in cell types, developmental phases, PH, incentive condition, experiment protocols, duration of treatment, stimulus, cellular environment and measuring methods.^16^, ^33^

It is still an open question whether AF autophagy plays a pro‐survival and pro‐apoptosis role during vascular remodelling. To date, the AFs autophagic conditions stimulated with FSP1 are still unknown. In our experiment, we found the levels of the autophagy‐related proteins (LC3B, beclin‐1 and Apg7) were significantly up‐regulated when treated with FSP1, whereas the level of p62 was significantly decreased. Meanwhile, the signalling molecules in RAGE, JAK/STAT and Wnt/β‐catenin pathways were synchronously and highly expressed. On the contrary, the results were reversed in the groups of siRNA‐FSP1, FSP1 + AG490, FSP1 + DKK, FSP1 + FPS‐ZM1 and FSP1 + Stattic (Figure 8). Transmission electron microscopy (TEM) analysis was used to assess cytoplasmic changes in AF cells. As a result, autophagic vacuoles were observed more in FSP1 group than those in siRNA‐FSP1 group (Figure 8). As expected, consistent with major reports, we deduced that FSP1 induce autophagy in AFs by the stimulating the crosstalk of RAGE, JAK2/STAT3 and Wnt3a/β‐catenin pathways. The findings of FSP1 promoting autophagy differed from those obtained by Hou S et al. Different cell types, microenvironment and stimulant conditions may account for the diverse effects of FSP1 on cellular autophagy.

Our previous research has shown that during the process of vascular remodelling, collagen is the main component of extracellular matrix (ECM) 28 and the subtypes I and III showed the highest expression levels in proliferative adventitia.^33^ Accumulating evidence has manifested that ECM modulation of autophagy often takes place in collagen synthesis or degradation in many types of cells.^34^, ^35^ However, no literature has been reported about the relationship between autophagy and collagen metabolism. In accord with other researches mentioned above, our study found that autophagic activation enhanced collagen (I and III) accumulation under the stimulation of FSP1 (Figure S6). Autophagy can provide material and energy in the process of organ or tissue remodelling.^34^ Therefore, we speculated that the process FSP1‐mediated autophagy may provide energy for the collagen synthesis in AF. However, the underlying mechanism about their correlation needs further study.

There were several limitations for this study. Firstly, no animal model has been provided to further validate our findings in vivo. Secondly, some autophagy‐related proteins, such as P62, incorporated into the mature autophagy and degrade in the autophagy, so the level of P62 is negatively related to autophagy. However, the detailed mechanisms by which FSP1 interacts with autophagy in AFs still need to be further investigated. Thirdly, there are many subtypes of signalling molecules in JAK/STAT and Wnt/β‐catenin pathways. Here, we have only studied some of them, which need to be further explored. Additionally, Wnt5a is one of the most highly studied non‐canonical members, whereas we have not explored it in this experiment.

To summarize, the data suggest our pivotal findings that FSP1 stimulates AF proliferation, adhesion, migration and autophagy through crosstalk among RAGE, JAK2/STAT3 and Wnt3a/β‐catenin signalling pathways. On the contrary, FSP1 knockdown accounts for the opposite results as mentioned above. Further studies are required to further delineate the biological function in vivo experiments. To our knowledge, this finding has not been previously reported in the literature. Therefore, our study may represent a novel therapeutic strategy for improving pathological vascular remodelling.

## CONFLICT OF INTEREST

All authors declare that they have no conflict of interest.

## AUTHOR’S CONTRIBUTIONS

Ping Liu designed experiments. Caihua Fu and Peilun Li carried out experiments. Caihua Fu and Wenhui Liu analysed experimental results. Xianwei Huang and Yansheng Liang analysed sequencing data and developed analysis tools. Caihua Fu and Ping Liu wrote the manuscript. All authors approved the final manuscript.
