# Corrigendum

**DOI:** 10.1111/jcmm.17774

**Published:** 2023-06-06

In the article by Caihua Fu et al.,^1^ there were errors in Figure 3 and Figure S3, the image of cell migration in FSP1 + siRNA‐FSP1 group after 24 h of stimulation was misplaced, resulting in the duplication of the image with the siControl group. In Figure S3, according to the original data records, the picture of cell migration after 12 h of stimulation in the FSP1 + FPS‐ZM1 group should be replaced. The authors confirmed that all results and conclusions of this article remain unchanged.

**FIGURE 3:**
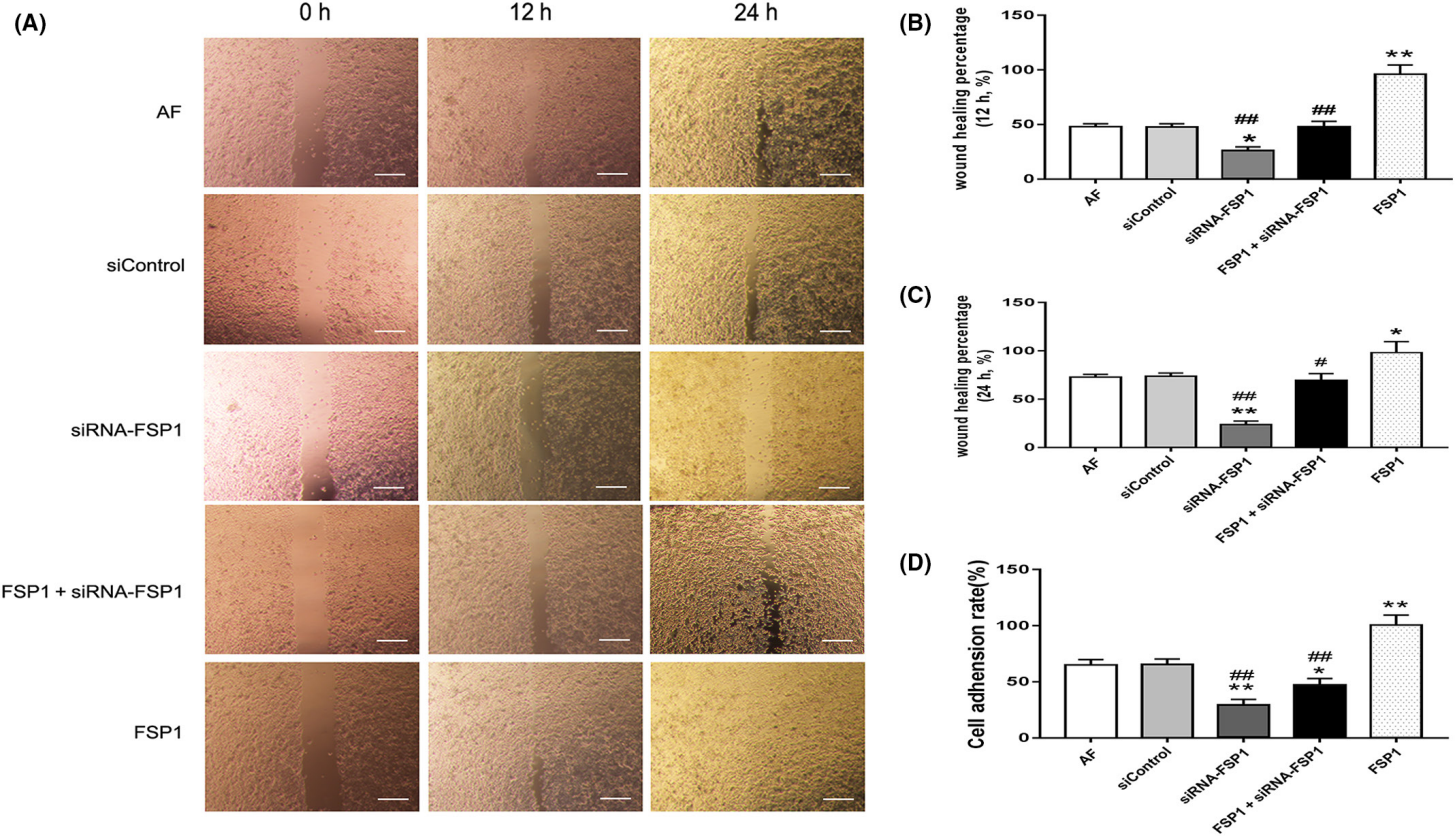
FSP1 promotes cell migration and adhesion. A, Wound healing test was observed at 12 and 24 h, respectively, after 40 nmol/L FSP1 stimulation. Scale bars represent 200 μm. Wound healing area was calculated by Image J. B, The comparison of 12 h' wound healing percentage. C, The comparison of 24 h' wound healing percentage. D, Cell adherent ability was calculated after 40 nmol/L FSP1stimulation for 24 h (data presented as mean ± SD; the comparison of multiple groups was performed by ANOVA; vs. AF group, *p < 0.05, **p < 0.01; vs. FSP1 group, ^#^ *p* < 0.05, ^##^ *p* < 0.01).
